# Rapid Detection of the H275Y Oseltamivir Resistance Mutation in Influenza A/H1N1 2009 by Single Base Pair RT-PCR and High-Resolution Melting

**DOI:** 10.1371/journal.pone.0021446

**Published:** 2011-06-24

**Authors:** Steven Y. C. Tong, Farshid Dakh, Aeron C. Hurt, Yi-Mo Deng, Kevin Freeman, Peter K. Fagan, Ian G. Barr, Philip M. Giffard

**Affiliations:** 1 Menzies School of Health Research, Charles Darwin University, Darwin, Northern Territory, Australia; 2 Infectious Diseases Department, Royal Darwin Hospital, Darwin, Northern Territory, Australia; 3 World Health Organization Collaborating Centre for Reference and Research on Influenza, Melbourne, Victoria, Australia; 4 Northern Territory Government Pathology Service Microbiology Laboratory, Royal Darwin Hospital, Darwin, Northern Territory, Australia; University of Hong Kong, Hong Kong

## Abstract

**Introduction:**

We aimed to design a real-time reverse-transcriptase-PCR (rRT-PCR), high-resolution melting (HRM) assay to detect the H275Y mutation that confers oseltamivir resistance in influenza A/H1N1 2009 viruses.

**Findings:**

A novel strategy of amplifying a single base pair, the relevant SNP at position 823 of the neuraminidase gene, was chosen to maintain specificity of the assay. Wildtype and mutant virus were differentiated when using known reference samples of cell-cultured virus. However, when dilutions of these reference samples were assayed, amplification of non-specific primer-dimer was evident and affected the overall melting temperature (T_m_) of the amplified products. Due to primer-dimer appearance at >30 cycles we found that if the cycle threshold (C_T_) for a dilution was >30, the HRM assay did not consistently discriminate mutant from wildtype. Where the C_T_ was <30 we noted an inverse relationship between C_T_ and T_m_ and fitted quadratic curves allowed the discrimination of wildtype, mutant and 30∶70 mutant∶wildtype virus mixtures. We compared the C_T_ values for a TaqMan H1N1 09 detection assay with those for the HRM assay using 59 clinical samples and demonstrated that samples with a TaqMan detection assay C_T_>32.98 would have an H275Y assay C_T_>30. Analysis of the TaqMan C_T_ values for 609 consecutive clinical samples predicted that 207 (34%) of the samples would result in an HRM assay C_T_>30 and therefore not be amenable to the HRM assay.

**Conclusions:**

The use of single base pair PCR and HRM can be useful for specifically interrogating SNPs. When applied to H1N1 09, the constraints this placed on primer design resulted in amplification of primer-dimer products. The impact primer-dimer had on HRM curves was adjusted for by plotting T_m_ against C_T_. Although less sensitive than TaqMan assays, the HRM assay can rapidly, and at low cost, screen samples with moderate viral concentrations.

## Introduction

During the influenza A/H1N1 2009 pandemic the widespread use of oseltamivir has been a key component of efforts to treat individual patients and provide prophylaxis for those at risk. Of concern there are now not only isolated reports of detection of oseltamivir resistant virus [1], [2], [3] but also evidence of emergence of oseltamivir-resistance during prophylaxis [4] and community clusters of cases [5]. To date, documented oseltamivir resistant influenza A/H1N1 2009 viruses carry a single nucleotide polymorphism (SNP) at position 823 (cytosine to thymine) of the neuraminidase gene which results in a histidine to tyrosine substitution at position 275 [6]. Detection of resistant virus is usually performed by phenotypic assays such as neuraminidase inhibition assays, or by sequencing of viral nucleic acid [7]. These assays are time consuming and often restricted to reference and research laboratories. In addition, they can lack sensitivity when there is insufficient viral concentration in clinical samples, or in the case of the phenotypic assay require a cultured isolate.

High-resolution melting analysis is an emerging technology that is based on monitoring the separation of double stranded DNA as the temperature is increased in the presence of DNA intercalating dyes. The advantages of HRM are that it is a single-step closed tube process incorporating the steps of reverse transcriptase and post-amplification analysis, and that it requires no reagents beyond real-time PCR master-mix and unlabelled oligonucleotide primers, so it is inherently simple and cost effective. HRM analysis of the neuraminidase gene has been used for typing of influenza [8] but not for the determination of oseltamivir resistance monitoring. The challenges for detecting the H275Y mutation are that the assay needs to be specific for the C to T SNP at position 823 and needs to take into account the potential impact upon melting curves of variation in starting RNA template quality and quantity in clinical samples. This report describes a SYBR green based real-time reverse transcription PCR (rRT-PCR) followed by HRM analysis to detect the H275Y mutation and the methodologies to address the challenges observed.

## Methods

### Ethics statement

The conduct of this study was approved by the Human Research Ethics Committee (HREC) of the Northern Territory Department of Health & Families and Menzies School of Health Research (HREC reference number 09/79). The HREC deemed that individual consent was not required from patients as there was no collection of identifying information in association with the samples used.

### Reference samples

Five oseltamivir sensitive influenza A/H1N1 2009 viral samples (A/Victoria/2048/2009, A/Victoria/2116/2009, A/Denmark/524/2009, A/Perth/184/2009, A/Brisbane/108/2009) and five samples that contained oseltamivir resistant (H275Y) virus (A/Osaka/180/2009, A/Perth/268/2009, A/Victoria/3132/2009, A/Denmark/528/2009, A/Perth/262/2009) were used as reference samples. Pyrosequencing and neuraminidase inhibition assays were used to detect the presence and relative mix of H275Y mutant strains [9]. To determine the specificity of the HRM assay for influenza A/H1N1 2009 we used five influenza A/H1N1 seasonal (non 2009) and three influenza A/H3N2 as negative control strains. RNA was extracted by the Roche MagNaPure LC protocol (Roche Diagnostics Australia, Castle Hill, NSW, Australia). The HRM assay was tested against 10-fold serial dilutions of extracted RNA from wildtype and mutant influenza A/H1N1 2009 samples and mixtures of mutant∶wildtype virus at 10∶90 and 30∶70 ratios. Starting concentrations of RNA were quantified by using absorption of light at 260 and 280 nm (A260/280) with a Biowave DNA spectrophotometer (Biochrom WPA, Blackburn, Victoria, Australia).

### Clinical specimens

We validated the HRM assay on 69 influenza positive clinical samples from the Royal Darwin Hospital. Samples were obtained from patients with an influenza-like illness between June 1 to August 30 2009 by nose and throat swabs. Swabs were placed in viral transport medium and RNA extracted by the Roche MagNaPure LC protocol. All samples were tested with TaqMan assays targeting the influenza A matrix gene and, if positive, subsequently targeting the haemaglutinin gene of influenza A/H1N1 2009 [10]. Of the 69 samples, 59 were influenza A/H1N1 2009, 3 were influenza A/H1N1 seasonal, and 7 were influenza A/H3N2.

### HRM assay design and protocol

We designed primers specific for influenza A/H1N1 2009 based on sequences of the NA gene downloaded from the National Center of Biotechnology Information from September 2009. A novel strategy of only amplifying a single base pair, the SNP at position 823, was chosen to maintain the specificity of the HRM assay. We were concerned that if the amplified region was extended, other sequence variations in the region of position 823 would non-specifically alter the melting temperature and melting curve. The primers were: H275Y F: 5′ GTCAAATCAGTCGAAATGAATGCCCCTAATTAT and H275Y R: 5′ GGATAACAGGAGCATTCCTCATAGT. The resulting product was predicted to have a lower melting temperature (T_m_) in the presence of a thymine (mutant) compared to a cytosine (wildtype) at position 823. These primers were predicted to form cross-dimers with a free energy (ΔG) of −10.09 (http://www.premierbiosoft.com/netprimer/index.html) and shortening or lengthening the primers did not appreciably reduce the likelihood of cross-dimer formation. We decided that the need for specificity of the HRM assay, and thus constraints upon primer positioning, would likely outweigh problems caused by potential primer-dimer formation.

We used a step-down PCR amplification method to improve specificity. Each reaction contained 5 µL QuantiFast SYBR Green RT-PCR Kit master mix (Qiagen, Doncaster, Victoria, Australia), 0.5 µM of each primer, 1 µL RT-mix and 1 µL RNA template in a final volume of 10 µL. The real-time PCR thermocycling parameters were: 50°C for 10 min, 95°C for 5 min; 4 sets of 2 cycles each of 95°C for 2 s and decreasing annealing temperatures from 68°C to 62°C in 2°C decrements for 6 s; 27 cycles of 95°C for 2 s, 60°C for 6 s; and 50°C for 20 s; followed by HRM ramping from 69°C to 79°C with fluorescence data acquisition at 0.2°C increments. The total reaction time was 62 minutes. Reactions were performed on a Rotor-Gene 6000 (Corbett Life Science, Concorde, NSW, Australia) instrument. These can no longer be purchased, but the QIAGEN Rotor-Gene Q devices are essentially identical.

### Statistical analysis

Raw data were exported from the Corbett Rotor-Gene 6000 software v.1.7 into Microsoft Excel 2007 (Microsoft, Redmond, Washington, USA) and Stata 10.1 (StataCorp LP, Texas, USA) for graphical and statistical analysis.

## Results

### Assay design and proof of principle using dilutions of reference samples

**Figure 1:**
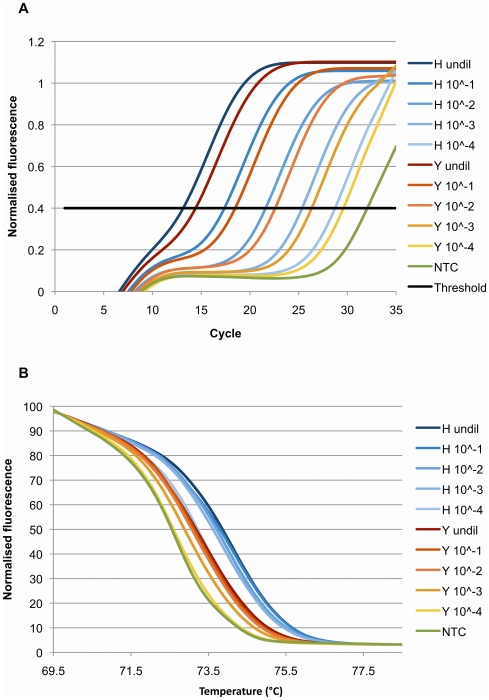
Relationship between cycle threshold and melting temperature. The relationship between cycle threshold (C_T_) and melting temperature (T_m_) demonstrated by ten-fold serial dilutions of wildtype (H) (A/Victoria/2048/2009) and mutant (Y) (A/Osaka/180/2009) virus samples. **A.** The C_T_ increases as the RNA template concentration decreases. The no template control (NTC) demonstrates late amplification after cycle 30, indicating the presence of primer-dimer. **B.** The T_m_ on the high-resolution melting normalized fluorescence graph decreases as the RNA template concentration decreases. The NTC melts at the lowest temperature. As the amplified product contains increasing proportions of the primer-dimer, the overall T_m_ is pulled towards that of the primer-dimer.

We initially tested the assay using dilutions of reference samples with wildtype (A/Victoria/2048/2009) and mutant (A/Osaka/180/2009) viruses. The cycle thresholds (C_T_) for the serial 10-fold dilutions were as predicted for such a series (**Fig. 1A**). It was also evident that non-specific primer-dimer amplification occurred as demonstrated by the presence of amplification products after cycle 30 in no-template controls and non influenza A/H1N1 2009 controls. Although the undiluted samples could be clearly differentiated based on the T_m_ and normalized fluorescence curves, there was overlap in the normalized fluorescence curves when comparing a 10^−4^ dilution of wildtype and the undiluted mutant containing sample (**Fig. 1B**). The presence of primer-dimer explains the downward T_m_ shift with reducing concentrations of template RNA (**Fig. 1B**). The primer-dimer product has a lower T_m_ compared to the desired amplification product. As the concentration of template RNA decreases, the proportional representation of end product due to primer-dimer increases, and therefore the overall T_m_ of the end product is decreased. Addition of Q solution (Qiagen Australia, Doncaster, Victoria, Australia) eliminated the detectable amplification of primer-dimers but significantly reduced the sensitivity of the amplification and resulted in less reproducible determination of the T_m_ values (data not shown). We concluded that the assay is more robust in the absence of Q solution and that the constraints upon primer placement made primer-dimer formation inevitable.

**Figure 2:**
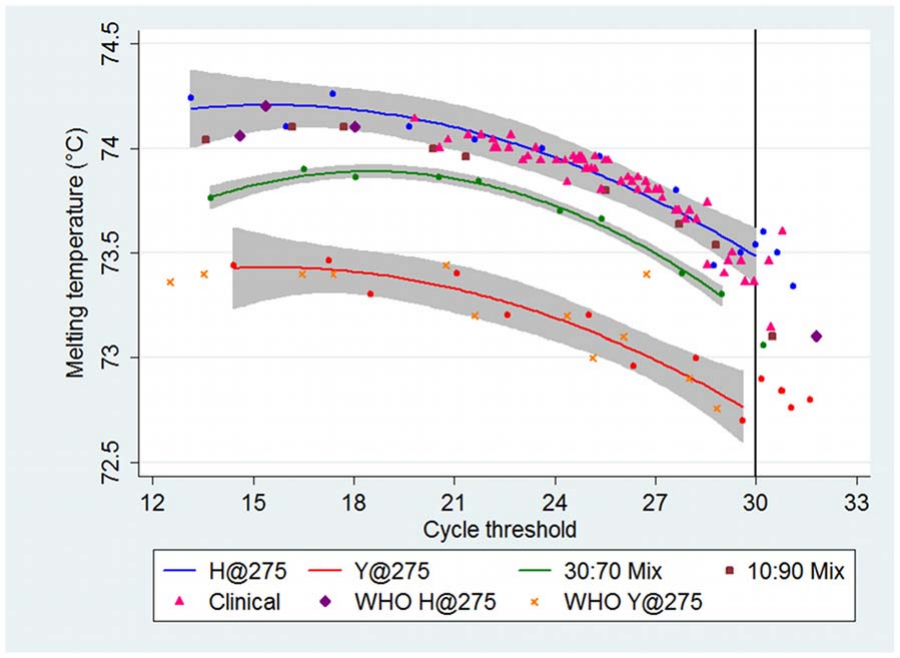
Plot of the melting temperature (T_m_) against the cycle threshold (C_T_). The blue, red, and green lines are the quadratic fitted lines with 95% confidence intervals for dilutions (blue, red and green circles) of the controls of wildtype (H@275) (A/Victoria/2048/2009), mutant (Y@275) (A/Osaka/180/2009), and 30∶70 mix of mutant∶wildtype respectively. The 10∶90 mutant∶wildtype consistently plotted below the wildtype fitted curve, but at the lower border of the 95% confidence interval. All 56 clinical samples that had a C_T_≤30 correlated with the wildtype virus (28 confirmed by pyrosequencing). Blinded WHO samples (four wildtype and four mutant containing) and dilutions of mutant virus (A/Denmark/528/2009 and A/Perth/262/2009) fit within the expected curves. One blinded sample (A/Perth/268/2009) plotted just below the 30∶70 curve and pyrosequencing determined this to be a 34∶66 mix of mutant∶wildtype. Raw data is provided in **Results S1**.

We therefore made allowance for the appearance of primer-dimer at >30 cycles by two means. We deemed that if the C_T_ for a sample was >30, the HRM assay was no longer able to consistently discriminate mutant from wildtype. Therefore the lower limit of detection of the assay was a 10^−4^ dilution of a starting RNA concentration of virus of 6.1 ng/µL that is the equivalent of 10^5^ gene copies/µL (**Fig. 1A**). We also noted the consistent inverse relationship between T_m_ and C_T_; samples with lower concentrations of RNA and a higher C_T_ also had a lower T_m_ (**Fig. 1A**). We adjusted for this by plotting T_m_ against C_T_. The fitted quadratic curves with 95% confidence intervals for wildtype and mutant containing samples, and also 30∶70 mutant virus mixtures, could now easily be discriminated (**Fig. 2**). The quadratic curve for the 30∶70 mix serves as a useful cut-off; points lying above this line will contain a minority of mutant virus, and points below this line will contain a significant proportion of mutant virus.

### Validation with additional defined reference samples

Using these methods, eight blinded reference samples were correctly called; three were wildtype and five mutant containing samples. This included one sample that plotted above the mutant curve but below the 30∶70 mixture curve. Pyrosequencing of this sample revealed a 34∶66 mutant∶wildtype mixture. The raw data of C_T_ and T_m_ of these reference samples is provided (**Results S1**).

### Validation with clinical sample material

We excluded clinical samples from the Royal Darwin Hospital that had an influenza A/H1N1 2009 Taqman detection assay [10] C_T_>35 as we considered that there would unlikely be enough RNA present in these samples for the H275Y assay. Subsequently we randomly selected 59 clinical samples that had been determined to have an influenza A/H1N1 2009 TaqMan detection assay C_T_ ranging from 22.52 to 32.95. The corresponding range of H275Y assay C_T_ was 19.8 to 30.77. Fifty-six clinical samples were predicted to be wildtype (**Fig. 2**), of which 28 were pyrosequenced and confirmed as such. Three samples (with TaqMan detection assay C_T_ of 29.81, 31.01 and 31.15) had H275Y assay C_T_>30 for which we considered the HRM assay would not be robust due to the poor amplification. The Pearson correlation coefficient for the C_T_ of the two assays was 0.92 (P<0.0001). Linear regression analysis predicted that samples with a TaqMan detection assay C_T_>32.98 would have an H275Y assay C_T_>30. We reviewed the TaqMan assay C_T_ for 609 consecutive positive clinical samples at the Royal Darwin Hospital and found that the HRM assay would be predicted to have a C_T_>30 in 34% (207 of 609) of clinical samples. All ten non influenza A/H1N1 2009 controls had a C_T_ of >30 cycles.

## Discussion

We have developed and evaluated a rRT-PCR HRM diagnostic assay to detect oseltamivir resistance due to the H275Y mutation in H1N1 2009 influenza viruses. Other RT-PCR assays to detect H275Y in influenza make use of various techniques including RT-PCR followed by restriction fragment length polymorphism [11], discrimination based on amplification curves with fluorescent TaqMan probes [12], [13], [14], [15], [16], [17], [18], a SYBR-green based rRT-PCR [19], rolling circle amplification [20] and a mismatch amplification mutation assay [21]. Advantages of the currently described HRM assay are its single-step, closed tube nature with post-amplification SNP interrogation, and the low cost of reagents. It is also rapid, with a total run time of 62 minutes. However, the key limitation to this HRM assay is that clinical samples with a low amount of viral RNA template cannot be reliably interrogated.

We have demonstrated two novel methodological approaches. First, we used PCR to amplify a single base pair with subsequent HRM analysis to ensure specificity of the HRM curves. Second, due to the constraints this placed on primer design, non-specific primer-dimer formation occurred and we successfully adjusted for the impact this had on the overall T_m_ of the reaction by plotting T_m_ against C_T_. Wildtype and mutant containing samples were therefore clearly discriminated up to a C_T_ of 30. The curve generated for 30∶70 mixed samples can be used as a cut-off to separate samples containing a significant proportion of resistant mutant virus; or alternatively, those samples with a T_m_ versus C_T_ value falling outside the confidence limits of the sensitive control curve could be targeted for further evaluation.

Our results were reproducible with runs on different days and with different operators (data not shown). Our experience and understanding is that the Rotor-Gene 6000 devices are extremely accurate with regards to relative temperature changes. However, the absolute temperature calibration may differ by up to 0.5°C between different machines. Therefore, we recommend initial calibration using samples of known mixtures of mutant and wildtype samples.

The key limitation of this assay is that clinical samples with a low amount of viral RNA template cannot be reliably interrogated. However, similar problems can exist for pyrosequencing and some rRT-PCR assays where up to 20% of clinical samples provided indeterminate results due to low viral load [17], [19]. Also, neuraminidase inhibition assays require the virus to be isolated in tissue culture or eggs before testing, something that is very problematic when samples have C_T_ values >30. Other molecular diagnostic assays that make use of labeled probes appear to be more sensitive with detection limits ranging from 2–500 gene copies/ml [13], [15], [18]. However, these assays are associated with the additional expense of labeled probes, and assays using TaqMan probes require two reactions per sample. A mismatch amplification mutation PCR assay that does not require probes has been described but also involves two reactions per sample as well as an additional gel electrophoresis step following the PCR reaction [21].

Given the simplicity (single reaction, single-step, closed-tube), low cost (single pair of unlabeled oligonucleotide primers) and rapidity of this HRM assay, we foresee a number of possible applications. It could be used to screen a large number of clinical samples that are known to contain sufficient amounts of virus. Similarly, culture stocks of virus where there is a large concentration of virus could be rapidly screened. Immunocompromised patients with persistent H1N1 09 infections and at risk of development of oseltamivir resistance have been reported to have low C_T_ values [22] and this HRM assay may be useful in detecting the H275Y mutation in virus from such patients. If there is an urgent clinical need to determine if resistance is present and the C_T_ value is too high, a repeat specimen using a more sensitive sampling technique such as a nasopharyngeal aspirate could be requested. As this assay can only confidently detect 30∶70 mutant∶wildtype mixtures, if a patient continues to shed virus despite appropriate treatment, performing the assay on sequential specimens should be considered to detect an increase in the mutant population to above this threshold. Finally, although this assay is specific for influenza A/H1N1 2009 alternative primer sets could be easily designed for seasonal H1N1 and H3N2 viruses.

## Supporting Information

Results S1 **Results for each sample with values for high-resolution melting (HRM) cycle threshold (C_T_), HRM melting temperature (T_m_), TaqMan H1N109 C_T_, pyrosequencing results with mutant %, and neuraminidase enzyme inhibition assay oseltamivir concentrations required to inhibit 50% of NA activity (IC_50_) (nm).** Blank cells indicate that an assay has not been performed.
(XLS)
